# Analyzing the impacts of global trade and investment on non-communicable diseases and risk factors: a critical review of methodological approaches used in quantitative analyses

**DOI:** 10.1186/s12992-018-0371-8

**Published:** 2018-05-24

**Authors:** Krycia Cowling, Anne Marie Thow, Keshia Pollack Porter

**Affiliations:** 1 Department of Health Policy and Management, Bloomberg School of Public Health, Johns Hopkins University, 624 N. Broadway, Hampton House 380A, Baltimore, MD 21205 USA; 2 Menzies Centre for Health Policy, School of Public Health, The University of Sydney, D17 Charles Perkins Centre, Sydney, NSW 2006 Australia

**Keywords:** Trade, Investment, Non-communicable diseases, Tobacco, Alcohol, Diet, Critical review

## Abstract

**Background:**

A key mechanism through which globalization has impacted health is the liberalization of trade and investment, yet relatively few studies to date have used quantitative methods to investigate the impacts of global trade and investment policies on non-communicable diseases and risk factors. Recent reviews of this literature have found heterogeneity in results and a range of quality across studies, which may be in part attributable to a lack of conceptual clarity and methodological inconsistencies.

**Methods:**

This study is a critical review of methodological approaches used in the quantitative literature on global trade and investment and diet, tobacco, alcohol, and related health outcomes, with the objective of developing recommendations and providing resources to guide future robust, policy relevant research. A review of reviews, expert review, and reference tracing were employed to identify relevant studies, which were evaluated using a novel quality assessment tool designed for this research.

**Results:**

Eight review articles and 34 quantitative studies were identified for inclusion. Important ways to improve this literature were identified and discussed: clearly defining exposures of interest and not conflating trade and investment; exploring mechanisms of broader relationships; increasing the use of individual-level data; ensuring consensus and consistency in key confounding variables; utilizing more sector-specific versus economy-wide trade and investment indicators; testing and adequately adjusting for autocorrelation and endogeneity when using longitudinal data; and presenting results from alternative statistical models and sensitivity analyses. To guide the development of future analyses, recommendations for international data sources for selected trade and investment indicators, as well as key gaps in the literature, are presented.

**Conclusion:**

More methodologically rigorous and consistent approaches in future quantitative studies on the impacts of global trade and investment policies on non-communicable diseases and risk factors can help to resolve inconsistencies of existing research and generate useful information to guide policy decisions.

**Electronic supplementary material:**

The online version of this article (10.1186/s12992-018-0371-8) contains supplementary material, which is available to authorized users.

## Background

When the United Nations adopted the Sustainable Development Goals as its guiding principles for global development through 2030, this included Goal 17, to “revitalize the global partnership for sustainable development” [1]. The specific targets comprising this goal identify the need for policy coherence to ensure global macroeconomic stability and sustainable development; one vital area for improved policy coherence is between the public health and international trade and investment sectors. Existing research establishes important links between these sectors [2–4]; however, additional evidence is needed to inform stronger trade and investment policies based on better understanding of their health implications.

Global flows of trade and investment are primary mechanisms through which globalization impacts health – both positively and negatively, including through social determinants of health such as poverty and inequality [5, 6], by altering working conditions and exposure to occupational risks [7], contributing to environmental pollution [8], and affecting the price and availability of health services and essential medicines [9, 10]. One subject area within this broader literature is the impact of global trade and investment policies on tobacco, alcohol, and dietary consumption, and resulting effects on non-communicable diseases (NCDs) [11]. Policies facilitating investment and trade in tobacco, alcohol, and nutrient-poor food and beverages can undermine individual- and community-scale interventions intended to reduce consumption of these products. Thus, trade and investment policies must be considered as points of intervention for combatting the growing global NCD epidemic and it is critical to examine the ways in which these policies shape consumption patterns and related health outcomes.

The objective of this study is to critically review the methods utilized in quantitative approaches to assessing the impact of global trade and investment policies on diet, tobacco, alcohol, and related health outcomes, and to develop recommendations and provide resources to guide future policy relevant research. To date, a relatively small but growing number of studies have used quantitative methods to investigate the impacts of global trade and investment policies on tobacco and alcohol use, diet, and related health outcomes. Several recently published reviews present partial syntheses of this literature, finding heterogeneity in results and a range of quality across studies. Conflicting findings may be in part attributable to a lack of conceptual clarity on these relationships and methodological inconsistencies [12, 13], warranting further examination of the theoretical underpinnings and analytical methods used in this body of research. Of particular concern is the lack of clear differentiation between trade and investment policies from their direct impacts (changes in trade and investment flows) and from the broader phenomena of economic globalization, of which these are key aspects. In an effort to identify all studies examining impacts of trade and investment policies, this review includes studies using any of these common alternatives as explanatory variables. Another key issue is endogeneity – an important concern given the many factors that can affect both trade and investment policies and population health outcomes, and the potential for bidirectional causal relationships. Burns, et al. provide one detailed illustration of these types of interrelationships [14].

This is the first review on these topics with a primary focus on quantitative methods, providing a point of reflection and identifying important ways to strengthen the conclusions and increase the policy relevance of future research in this area. This study builds upon other recent reviews with a unique emphasis on key NCD risk factors and related health outcomes and identifies many studies not included in any existing reviews. In addition to posing novel methodological questions of this literature, we assess the consistency of conclusions across previous reviews and examine the extent to which these conclusions hold for this expanded set of studies.

## Methods

### Study design

This study is a critical review, distinguished from other types of reviews by an aim to go “beyond mere description of identified articles and includ[e] a degree of analysis and conceptual innovation,” leading to a “starting point for further evaluation” [15]. Selected research questions in this study overlap with those in other recent reviews, however, this is warranted due to the unique scope of this study, which encompasses many articles not included in previous reviews. The following research questions guided this analysis of studies examining the impacts of global trade and investment on diet, tobacco, alcohol, and related health outcomes, to date:

1. What study designs have been used?
2. What data sources have been used?
3. What indicators of trade and investment have been used?
4. What health outcome and risk factor indicators have been used?
5. What confounding, mediating, and moderating variables have been examined?
6. What are the strengths of the data and methods used?
7. What are the limitations of the data and methods used?
8. What lessons can be drawn from the existing literature, to inform future policy relevant research?

The methodological approach to identify existing literature was a review of reviews, which provided both an efficient means to identify relevant articles and an opportunity to explicitly complement reviews focused on primarily findings with our exclusive focus on methods.

### Literature search

To identify review articles, we searched the following databases encompassing peer-reviewed and grey literature in health, economics, and social sciences: PubMed, EMBASE, EconLit, Scopus, CAB Direct, Web of Science, Cochrane Library, PAIS Index, and ProQuest Dissertations and Theses. The specific search terms used are provided in additional file 1. We considered all article types, including reports, conference presentations, and graduate work; results were limited to those available in English and published in 2000 or later, due to the relatively recent increase in studies published on these topics.

**Fig. 1:**
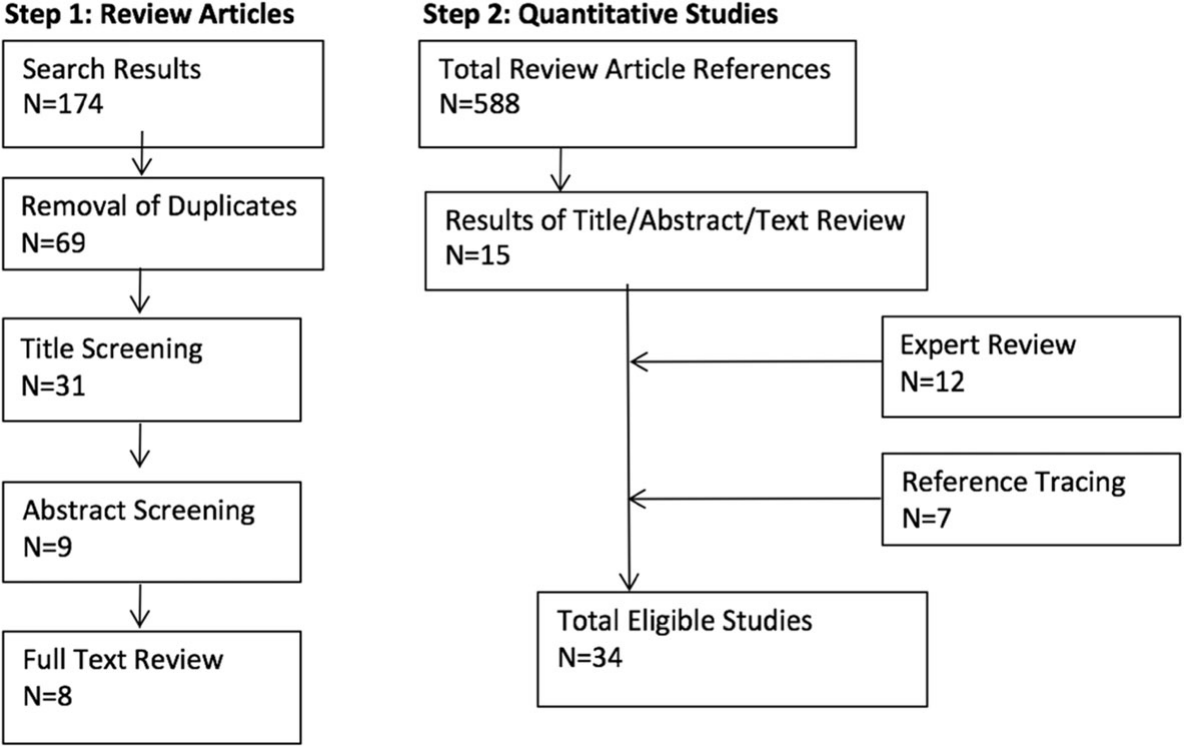
Results of literature search and screening process

This search yielded 174 total results; 69 were unique. After an initial screening of titles for relevance, 31 items were kept for abstract review, resulting in nine items for full text review; six review articles met our inclusion criteria (Fig. 1). The inclusion criteria for review articles were as follows: 1) self-described as one of the following: systematic review, literature review, synthesis of literature, or qualitative literature review, 2) the inclusion criteria used in the review captured studies examining the impacts of any aspect of trade or investment, or broader related topics (e.g., globalization, macroeconomic reforms), on one or more of: diet or nutrition; tobacco use; alcohol use; or related health outcomes. These reviews were not limited to those with a focus on quantitative literature, although we only extracted quantitative studies from the reviews. Two review articles [12, 16] contained a reference to another relevant review [17, 18], for a final sample of eight reviews.

We identified quantitative studies captured by each review from lists of included studies in manuscripts or appendices, if provided. We requested these results from review authors not providing such lists in published materials (*n* = 4); however, none provided these and two directed us to the reference lists, so we screened the citations of all review articles. There were a total of 588 references, with substantial duplication. Title screening, abstract review, and full text review were used, as needed, to identify studies matching our inclusion criteria: 15 eligible studies were identified through this process. Additional studies were identified from expert review of this list (the authors plus two external experts) (*n* = 12) and from reference tracing from eligible studies (*n* = 7). A total of 34 quantitative studies were included.

The inclusion criteria for quantitative studies were as follows: 1) use of quantitative analysis, which included a statistical test or model and was not purely descriptive, 2) examination of any aspect of international trade or investment, or a broader related topic (e.g., globalization) as the exposure of interest, 3) examination of one or more of the following outcomes: tobacco, alcohol, or dietary consumption, or related health outcomes (either morbidity or mortality). All studies examining adult mortality or life expectancy were included as these are definitively impacted by diet, tobacco, and alcohol use. Studies examining only infant or child mortality were excluded as beyond the scope of this study because these are more indirectly impacted by changes in these NCD risk factors, which predominately affect adult health. Articles were restricted to those available in English; all article types were included and no restrictions were placed on the year of publication.

### Quality assessment

Three of the eight review articles we identified assessed study quality. Key conclusions are presented here and were used as a starting point to develop a new quality assessment tool for this study (see Additional file 2). Existing quality frameworks were considered, but none were sufficiently tailored to these topics. One previous review also determined that existing quality assessment tools were not adequately suited to this literature and elected to develop a new tool [13]. The tool developed for that study assessed traditional measures of quality including the reliability of data, strength of analysis, and presentation of results, providing substantial detail but a more generic assessment. For this study, we opted to develop a simpler and more focused quality assessment tool to provide an evaluation tailored to applied research in this area, the development of which was heavily informed by the findings and conclusions of the eight review articles. This was designed to specifically assess common weaknesses identified by previous reviews and evaluate the conceptual basis for and appropriateness and consistency of data sources and indicators for different research questions. This encompasses: whether trade and investment indicators align with the aspect of trade or investment being investigated, the specificity of explanatory variables, the choice of confounding variables considered, and the relevance of data sources utilized. In addition, this tool incorporates selected traditional components of study quality, including control for confounding, and inclusion of sensitivity analyses.

## Results

### Review articles

Table 1 displays key characteristics of the eight review articles. All reviews searched multiple peer-reviewed databases and all but one [16] also searched grey literature. The reviews differed in the degree of specificity to the scope and research questions guiding this review. As a result, not all identified a large number of quantitative studies relevant to this review, but all provided insights into aspects of the existing literature that can inform future research. Across the reviews, several themes emerged regarding weaknesses of methods used in studies to date and areas for development.

**Table 1:** Review articles: characteristics and key conclusions

Author (Year)	Scope/inclusion criteria *(search date range)*	Number of studies identified*	Relevant conclusions regarding existing literature
Breman & Shelton (2007) [17]	Structural adjustment programs (SAPs) and health outcomes; emphasis on empirical analyses *(dates not specified)*	76	- Three main policies of SAPs have been the focus of this literature: reduced government expenditures, liberalized markets, and exchange rate devaluation - “Overwhelming majority” of studies portray the impacts of SAPs on health as negative, but among strictly empirical studies, approximately even split between findings of positive, negative, and neutral impacts
Young, et al. (2009) [66]	Globalization and co-morbidity between infectious and chronic disease *(1950 – end date not specified)*	Not specified	- This review technically met our inclusion criteria but the globalization aspect was very minor in the results/discussion
Loewenson, et al. (2010) [19]	Globalization and nutritional outcomes in sub-Saharan Africa *(1990–2009)*	199	- Limited empirical work in Africa - Need for more research on gender dimensions of globalization and health
Friel, et al. (2013) [18]	Studies that developed approaches, methods, or indicators to monitor impacts of trade agreements on food environments from an obesity/NCD perspective; examined impacts of trade agreements on food chains and the food environment; or conceptualized links between trade liberalization and food environments *(1990 – January 2013)*	9	- “No studies were identified which used methods or indicators to systematically monitor trade agreements through an obesity/NCD lens” - Proposes potential indicators and food categories for monitoring the impacts of trade agreements on national food systems and food environments
Baker, et al. (2014) [67]	Trade liberalization, non-communicable diseases, and risk factors in Asia *(dates not specified)*	Not specified	- Understanding of the mechanisms linking transnational corporations and increased consumption of tobacco, alcohol, and unhealthy foods and beverages “appear to be theoretically and empirically underdeveloped in the public health literature”
Burns, et al. (2016) [13]	Quantitative studies investigating the relationship between international trade or foreign direct investment, and non-nutritional population health outcomes *(until end of 2014)*	16	- Current evidence on FDI as determinant and consequence of health is unclear; more research needed - Sample stratification may critically affect the estimated relationship between trade and health in international panel studies (e.g., nature of goods imported/exported, industry of international investments, position in global supply chain) - Important to consider mutual association when analyzing trade or FDI and health; adjustments for reverse causality were “typically crude” or absent - Surprisingly limited use of individual-level data
Barlow, et al. (2017) [22]	Quantitative studies of the health impacts of trade and investment agreements or policy. *(1960 – January 2016)*	17	- “Trade and investment measures varied in specificity” - Studies with stronger methodological designs most often used trade indicators with weak specificity - Mechanisms mediating links were seldom explored - Strong reliance on country-level data precludes exploration of social groups where effects are concentrated
McNamara (2017) [16]	Studies explicating a clear analytical framework for conceptualizing pathways between trade liberalization and health *(until end of 2015)*	43	- “Many authors include financial flows and foreign investment within conceptualizations of trade liberalization” - “Trade liberalization itself is seldom explicitly defined in frameworks”

*Number of studies specified by the authors as meeting the inclusion criteria of the review, not the number of references

One important area of consensus was that many studies do not clearly define the aspect of trade or investment being investigated and that explanatory indicators are often not sufficiently specific. Emphasizing the challenges posed by inconsistent definitions and indicators, both reviews focused on quantitative studies [12, 13] were unable to conduct meta-analyses due to heterogeneity “in measurement methods, research designs, and outcome variables” [12]. A second theme was the lack of exploration of mechanisms linking trade and investment with risk factors and health outcomes. A third area of consensus was the need to increase the use of individual-level data and assess impacts by individual-level characteristics. Loewenson highlights the particular importance of understanding the gender dimensions of globalization’s impacts [19], which requires data disaggregated by gender.

### Quantitative studies

Of 34 quantitative studies examining the relationship between some aspect of global trade or investment and tobacco, alcohol, diet, or related health outcomes, 18 examined at least one NCD-related health outcome, either morbidity or mortality. Ten studies examined changes in one or more aspects of dietary intake and nine studies considered average body mass index (BMI) or the prevalence of overweight or obesity. Tobacco and alcohol consumption were assessed by only three studies and one study, respectively. In ten studies, the stated exposure of interest was globalization; seven studies used a broad similar exposure such as “market deregulation” or “economic freedom”; 15 studies had a primary focus on some aspect of trade; and five studies examined investment, although how each of these was conceptualized and quantified was highly variable and inconsistent across studies. (Several studies used multiple exposures and/or outcomes).

The vast majority of studies (*n* = 29) examined associations over time using longitudinal data; only five studies used strictly cross-sectional data. Most studies examined a large number of countries (mean sample size was 64 countries) over one or more decades (on average, data spanned 23 years, ranging from 1960 to 2014). Three studies used natural experiment designs [20, 21], with one employing synthetic controls [22]. Nearly all studies reported using fixed effects regression models; exploration of random effects was infrequently reported (*n* = 4) [23–26]. Two studies used path analysis [27], one with structural equation modelling [28]. Five studies used instrumental variables [14, 29–31], one with a gravity model [32]. Tables 2, 3, and 4 display selected characteristics of these 34 studies. Additional details by individual study are provided in an additional file (see Additional file 1).

**Table 2:** Trade and investment indicators used in quantitative studies

Indicator	Number (%) of studies using as explanatory variable
KOF Globalization Index (Swiss Federal Institute of Technology)	9 (26)
FDI inflows/GDP *(%)*	8 (24)
Total trade (imports + exports)/GDP *(%)*	8 (24)
Entry into force of a specific agreement or SAP *(indicator variable)*	5 (15)
CSGR Globalisation Index (Univ. of Warwick)	2 (6)
Economic Freedom of the World Index (Fraser Institute)	2 (6)
Mean applied tariff rate	2 (6)
Average imports, weighted by partner countries’ infant mortality rates	1 (3)
Black market premium	1 (3)
Imported food/total food *(%)*	1 (3)
Index of Economic Freedom (Heritage Foundation/WSJ)	1 (3)
Index of service sector liberalization (World Bank)	1 (3)
Maastricht Globalisation Index (Univ. of Maastricht)	1 (3)
Sachs-Warner Index *(indicator variable)*	1 (3)

Counts and percentages do not sum to the total number of studies reviewed (100%) because some studies used multiple explanatory indicators

**Table 3:** NCD-related health outcome and risk factor indicators used in quantitative studies

Category	Indicator	Number (%) of studies using as outcome variable
*Health outcomes*	Life expectancy (total and/or by sex)	14 (41)
	Adult mortality rate (probability of death between ages 15 and 60)	3 (9)
	CVD mortality rate	1 (3)
	Diabetes prevalence	1 (3)
	NCD mortality rate	1 (3)
	Proportion of deaths attributable to CVD	1 (3)
*Over-nutrition*	Mean BMI (adults, total and/or by sex)	5 (15)
	Obesity prevalence (total and/or by sex)	4 (12)
	Overweight prevalence (total and/or by sex)	2 (6)
*Diet*	SSB imports/sales per capita	3 (9)
	Consumption per capita for selected food groups (e.g., animal proteins, sugars)	2 (6)
	Average caloric intake	1 (3)
	Consumption of ‘unhealthy’ foods (% of total spending/caloric intake)	1 (3)
	Supply of caloric sweeteners per capita	1 (3)
	Ultra-processed products sales per capita	1 (3)
*Tobacco*	Cigarette consumption per capita	2 (6)
	Tobacco sales per capita	1 (3)
*Alcohol*	Alcohol sales per capita	1 (3)

Counts and percentages do not sum to the total number of studies reviewed (100%) because some studies used multiple outcome indicators

**Table 4:** Country-level confounding variables controlled for in two or more studies

Indicator	Number (%) of studies using as confounding variable
GDP (or GNI) per capita (including squared term or growth rate)	26 (76)
Urbanization rate (or urban growth rate)	10 (29)
Population (total, density, or growth rate)	7 (21)
Educational attainment (years completed, enrollment rate, or literacy rate)	6 (18)
Income inequality	4 (12)
Female labor force participation rate	3 (9)
Health expenditure (% of GDP, total or public)	3 (9)
Average caloric intake	2 (6)
Consumer price index	2 (6)
Dependency ratio	2 (6)
FDI (total or % of GDP)	2 (6)
Fertility rate	2 (6)
Political rights/civil liberties index	2 (6)
Polity score	2 (6)
Immunization rate (any type)	2 (6)
Smoking prevalence	2 (6)
No confounders included/tested in models	7 (21)

Counts and percentages do not sum to the total number of studies reviewed (100%) because most studies included multiple possible confounders

As shown in Table 2, the three most frequently used explanatory variables were the KOF Globalization Index (reflecting economic, social, and political dimensions of globalization) [33]; total trade (sum of imports and exports) relative to GDP; and total FDI inflows as a percent of GDP. The World Bank World Development Indicators (WDI) database was the most commonly cited source for estimates of trade and investment flows. Authors using the KOF Globalization Index, or one of four other multifaceted indices [34–37], all elected to parse out a subcomponent most relevant to trade and investment. A smaller number of studies used binary indicators to distinguish the time period before and after entry into force of a specific agreement (World Trade Organization [21], North American Free Trade Agreement [22], bilateral U.S. free trade agreement [20], and Section 301 of the U.S. Trade Act [38]) or successful implementation of a structural adjustment program (SAP) [39]. Across nearly all studies (*n* = 30), explanatory indicators reflected economy-wide attributes, as opposed to sector-specific indicators more relevant to the outcomes investigated, as used in only four studies [14, 40–42].

Table 3 displays the NCD-related health outcome and risk factor indicators used in these quantitative studies. The most frequently used indicators were life expectancy at birth and mean BMI. A wide range of dietary indicators were used across studies; sugar-sweetened beverages (SSBs) have received the greatest focus within this literature. Three studies examined SSB imports or sales [20, 21, 41] and additional studies explored these indirectly through their contribution to consumption of sugars, caloric sweeteners, and ultra-processed products. Tobacco consumption was measured using cigarette consumption or tobacco sales per capita; alcohol consumption was measured using alcohol sales per capita.

Outcome variables were most often constructed from three sources of country-level information: World Bank WDI, Euromonitor International Global Market Information Database, and the Food and Agriculture Organization (FAO). Only four studies used individual- or household-level data from national health, consumption, or expenditure surveys [24, 42–44].

Table 4 presents country-level confounding variables used in two or more studies. Individual- and household-level confounders are excluded due to the small number of studies examining data at these levels; confounders explored in only one study are not listed because many of these do not have wider applicability. The most frequently used confounder was a measure of economic size (*n* = 26), most often GDP per capita. Other common confounding variables were the percent of the population living in an urban area or the urban growth rate (*n* = 10); a measure of population, either total, density, or the growth rate (*n* = 7); an indicator of educational attainment, either average years of school, enrollment rates, or literacy (*n* = 6); and income inequality, typically the Gini coefficient (*n* = 4). Seven studies did not include any confounding variables in any model specifications.

Only 6 studies used statistical approaches to investigate possible mechanisms of broader relationships. One study examined fast food transactions as a mediator between market deregulation and mean BMI, and in addition, examined total caloric intake, animal fat, and soft drink consumption as mediators between fast food transactions and BMI [30]. Two studies explored economic inequality as a mediator for globalization – one for effects on life expectancy [27] and one for impacts on mean BMI [26]. One study each examined: FDI inflows as a mediator between joining a U.S. FTA and SSB sales [20]; overweight prevalence and tobacco use as mediators between trade and investment policies and CVD mortality [28]; and GDP per capita, the measles immunization rate, and government health expenditures as mediators between trade flows and life expectancy [32].

Among studies using national-level data, the predominant moderating variable was country income level – either GDP per capita or a categorical variable for high-, middle-, and low-income. Four studies explicitly included one of these measures as a moderator in regression models (either through stratification or an interaction term) [29, 45–47]. In addition, many studies used a sample of countries of a limited income range (e.g., OECD countries), implicitly exploring relationships which may differ from those in countries at different levels of national wealth. A few additional moderating variables were considered by only one or two studies. One study assessed whether there were differences in the association between economic freedom and BMI among “market liberal” countries (i.e., U.S., U.K., Canada, and Australia) versus others [25]. Another examined world region as a moderator between successful implementation of a SAP and life expectancy [39]. Another using BMI, by sex, as the outcome explored gender as a moderator of the relationship with globalization [26]. A high level of political rights was explored as a moderator between economic freedom and life expectancy in one study [48]. Finally, two studies created categorical versions of either the explanatory or outcome variable to examine differences in the relationship between globalization and overweight or obesity prevalence [23, 43]. One study also created categories from outcome variable values to examine differences in the relationship between globalization and the food supply [49].

Studies using individual- and household-level data were more likely to examine moderating factors. One study using household-level data explored urban versus rural residence as a moderator between the proportion of food imported and consumption of “unhealthy” items [42]. Another used the interaction of gender and urban/rural location to explore differences in the relationship between macroeconomic factors and BMI [24]. A study on dietary patterns following the opening of South Korea’s food industry to the global economy examined differences in consumption by age group and sex [44].

#### Strengths and weaknesses of study designs

Two of the eight review articles were focused exclusively on quantitative studies and included assessments of study quality, reaching similar conclusions that the overall quality of this evidence is moderate. Through application of our quality assessment tool to 34 studies, we confirmed this overall conclusion and identified additional strengths and weaknesses of this literature.

Across studies, a key strength was the inclusion of sensitivity analyses, which demonstrate the reliance of study conclusions on specific methodological choices. Most studies (*n* = 28) reported at least one sensitivity analysis and/or described the robustness of findings to alternative model specifications. However, there was substantial variability in the degree to which studies explored and described these variations. The strongest studies provided multiple model specifications, for example, with and without selected confounding variables, as well as reported the results of sensitivity analyses, such as varying the set of countries included in the sample or altering the construction of outcome variables.

Another key strength, specific to the 13 studies that used a globalization or macroeconomic index as an explanatory variable, was disaggregation of the index to assess a component most relevant to trade or investment. For example, many authors using the KOF Globalization Index examined the economic dimension separately from the social and political dimensions (e.g., [28]). For analyses intending to examine trade or investment as the exposure of interest, such disaggregation generates explanatory indicators better aligned with the research questions by focusing specifically on economic globalization.

A key weakness of these studies was a lack of clarity about the aspect of trade or investment being explored, often regarding its precise definition as well as its relationship to the indicator used to reflect it. Across studies, the same indicators were used to represent different constructs. Total trade relative to GDP was used as a measure of trade openness [45, 50], trade liberalization [29, 47], and economic globalization [51]; FDI was used as a measure of globalization [46] and “market integration” [52]. The various globalization and macroeconomic indices conflate trade and investment, precluding any disentanglement of these effects. Only four studies considered both trade and investment as separate explanatory indicators [20, 23, 24, 50].

One challenge of many longitudinal analyses is the possibility of endogeneity, or reverse causality. Only one of the reviews discussed the need to better account for reverse causation, which the authors noted many studies had not even attempted to address [13]. Of 29 quantitative studies using longitudinal data, ten mentioned any use of methods to assess or control for endogeneity through the study design or statistical models. Studies that did so approached this in a variety of ways – most included lagged independent variables in regression models (e.g., [53, 54]), others used instrumental variables [14, 31] or switched the independent and dependent variables to examine the presence of any measurable relationship in the opposite direction [45].

Another key issue with longitudinal data is the likelihood of autocorrelation between repeated observations for the same country (or individual or household). 13 of 29 studies using longitudinal data described some adjustment for autocorrelation in statistical models, through a variety of different means: robust standard errors, use of lagged dependent variables as predictors, or by imposing correlation structures on model residuals.

### Inventory of data sources

As a resource for future research, an inventory of useful data sources for measuring trade and investment, identified from these studies, is presented in Table 5. These are supplemented with additional data sources known to the authors. Data are organized by the aspect of trade or investment (policy, liberalization, flows) measured by each, to encourage the use of data and indicators appropriately aligned with research questions. Furthermore, only sources with relatively specific data are included in the table, which can be considered in place of more generic indicators that have been frequently utilized, e.g., total trade flows relative to GDP.

**Table 5:** Detailed data sources for trade and investment policies, liberalization, and flows

Topic	Measure	International data sources
*Policy*	Treaty membership	• WTO membership database • UNCTAD International Investment Agreements Navigator
	Depth of commitments in specific agreements	• Design of Trade Agreements (DESTA) project • Mapping BITs
	Presence and outcome of trade or investment disputes	• WTO Dispute Settlement Gateway • UNCTAD Investment Dispute Settlement Navigator
*Liberalization*	Product-specific tariff rates	• UNCTAD TRAINS database • WTO Tariff Download Facility
	Non-tariff measures	• UNCTAD TRAINS database • USDA Foreign Agricultural Service reports • UNCTAD International Investment Agreements Navigator
*Flows*	FDI, by sector & industry	• International Trade Center database • UNCTADstat *(by request)*
	Product-specific imports & exports	• UN Commodity Trade Statistics (UN Comtrade) • World Bank World Integrated Trade Solution (WITS) • FAO Food and Commodity Balance Sheets • Index Mundi • USDA Foreign Agricultural Service
	Retail sales	• Euromonitor Global Market Information Database

## Discussion

This study examined eight review articles and 34 quantitative analyses of relationships between global trade and investment and diet, tobacco, alcohol, and related health outcomes. This is the first analysis with a primary focus on methodological approaches to investigating this topic, providing practical guidance and resources for future research. Several important weaknesses were identified in these quantitative studies: poorly defined exposures; mechanisms not sufficiently explored; inconsistency in the choice of confounding variables; and autocorrelation and endogeneity often not accounted for in longitudinal analyses. The inventory of explanatory and outcome variables and identified gaps in this literature suggest priorities for future work and possible ways to construct analyses. Citations for studies with different characteristics provide examples of design or analysis features that other researchers may be interested in applying. Lastly, the inventory of data sources classifies these in a way that enables each to be appropriately aligned with indicators and research questions.

### Opportunities for further public health research on trade and investment

This analysis revealed important gaps in the literature from a public health perspective and highlights opportunities for further research. Alcohol use has been evaluated by only one study. No studies have used the *prevalence* of tobacco or alcohol use as outcomes (only examining sales or consumption). Another area for additional research is childhood obesity, an important determinant of NCDs in adulthood [55] that has not been examined in the context of global trade and investment policies. Childhood obesity may be affected by trade-related increases in consumption of infant formula or nutrient-poor food, as suggested by existing studies [56, 57]. Finally, few studies have examined morbidity due to specific NCDs or NCD-related mortality, as opposed to the relatively frequent exploration of life expectancy and all-cause mortality as outcomes.

Future public health research would also benefit by separating trade and investment as explanatory variables, and further consideration of the role of investment. It is likely that trade and investment impact on public health outcomes through different mechanisms. For example, while key effects of liberalized trade may occur through increased imports, the impacts of liberalizing investment may operate through increases in local production [58]. Another area unexplored to date is comparing effects of different trade and investment agreements based on the depth of commitments, which requires quantifying commitments for statistical analysis. Two sources provide this type of data: the Design of Trade Agreements (DESTA) database [59] (for trade agreements) and Mapping BITs [60] (for investment treaties). A related challenge is the difficulty of quantifying non-tariff measures, for which a range of alternative techniques are available [61].

### Methodological lessons for future research

This analysis indicates that future research on the impact of trade and investment on public health outcomes would be strengthened by: 1) clearly defining exposures of interest; 2) exploring mechanisms of these relationships through analysis of mediating variables; and 3) increasing use of individual-level data for assessing public health impacts. Additional ways to improve the robustness of future studies were also identified: developing consensus and consistency in the choice of key confounding variables; utilizing more sector-specific versus economy-wide trade and investment indicators; testing and adequately adjusting for autocorrelation and endogeneity when using longitudinal data; and presenting results from alternative statistical models and sensitivity analyses. The implications of these findings for future research is elaborated below.

First, future studies on these topics should explicitly delineate the aspect of trade or investment being explored, i.e., whether a particular *policy*, the degree of *liberalization*, or *flows* of goods or capital is the exposure of interest, as distinguished in Table 5. Without agreement on these definitions and the indicators used to reflect each, it will remain difficult to synthesize and compare findings across studies. While useful for other purposes, it is difficult to generate specific policy recommendations from aggregate indicators of globalization or economic freedom, yet these have been used frequently as explanatory variables. Studies using such indices as explanatory variables and life expectancy or all-cause mortality as outcomes may be least informative for policy, due to the lack of specificity in both predictors and outcomes and the myriad of possible confounding factors. It is arguable whether any additional studies of these types are needed and researchers are encouraged to consider whether more nuanced and specific research questions may produce more actionable information.

Second, there is a need for additional research that explicitly explores mechanisms linking global trade and investment to NCD risk factors and health outcomes. Several published conceptual frameworks illustrate hypothesized mechanisms of these relationships and can provide a starting point for designing such analyses [11, 18, 28, 62]. Furthermore, several published qualitative and descriptive studies provide detailed examples and explore the evidence to support one or more of these mechanisms [58, 63–65]. The quantitative study by DeVogli that examines market deregulation, fast food consumption, soft drink, animal fat, and total caloric intake, and mean BMI, provides a useful example of an investigation of a cascade of events with presumed causal connections [30]. More nuanced explorations of this sort will generate more actionable information for policy decisions.

Third, greater use of individual-level data can facilitate identification of subpopulations where health impacts are concentrated, critical considering that the economic benefits of trade and investment are known to be unevenly distributed. Greater reliance on household- and individual-level data may also help to fill other research gaps, particularly regarding NCD morbidity and the prevalence of tobacco and alcohol use as outcomes.

Fourth, a broad range of confounding variables are inconsistently used across studies, including controlling for factors that are elsewhere used as exposures or outcomes. This suggests the need for research firmly grounded in a conceptual model, such as the frameworks referenced above, illustrating mechanisms and factors influencing hypothesized effects. The degree to which theoretical and empirical research on these topics may be siloed is illustrated by the results of the review by McNamara, for which the inclusion criteria specified that studies “explicat[e] a clear analytical framework for conceptualizing pathways between trade liberalization and health” [16]. No quantitative studies were identified from that review – it is significant that studies providing a strong conceptual basis for these links and those including quantitative analysis are so far mutually exclusive.

Fifth, the finding that only six studies to date have used any type of sector-specific, as opposed to economy-wide, indicators, suggests an opportunity for new research that is more nuanced and informative. However, a key challenge is the paucity of data. Mendez, et al. provide arguably the best analysis of sector-specific data to date, using product-specific applied tariff rates, but acknowledge their analysis would be strengthened with FDI data by sector, which were not available [41].

Finally, principles of study quality that apply to longitudinal analyses more generally are pertinent to this literature: as evident in the 34 quantitative studies reviewed, country-level panel data are the most commonly used for investigating these topics. All studies using longitudinal data should assess the presence of autocorrelation and adjust for this when needed, to ensure significance tests are valid. Failing to adjust for correlations between repeated observations over time can lead to biased standard errors, possibly leading to incorrect conclusions about study hypotheses. Furthermore, the possibility of reverse causality should also be considered and accounted for in the study design and/or analysis. For example, increasing consumer demand for tobacco or alcohol may attract investors rather than investments in these industries preceding consumer demand. Verifying the consistency of pre-intervention trends in dependent variables can identify this potential problem in comparative interrupted time series models; alternatively, study designs such as instrumental variables can be explored. Finally, given the many methodological choices required, the substantial potential for confounding, and the inconsistency of findings from past research, it is critical to include sensitivity analyses and assess the robustness of findings to model specification to accurately portray the certainty of study conclusions.

### Limitations of this review

Important factors may limit the findings and conclusions of this review. First, there may be additional studies meeting the inclusion criteria for either review articles or quantitative studies that were not captured by the selected search strategies. We attempted to minimize this possibility by using multiple databases to identify review articles and multiple search methods to identify quantitative studies. A greater than expected proportion of the quantitative studies were identified through expert consultation or reference tracing, rather than from review articles. A few explanations are possible: recent quantitative studies were published outside the dates searched by previous reviews; the scopes of the review articles did not precisely align with the present study; search methods of previous reviews may not have been sufficiently interdisciplinary. At the review article screening stage, the vast majority of articles were excluded due to an absence of statistical analysis; this literature is predominantly comprised of qualitative analyses and articles presenting strictly descriptive data. Second, publication bias may affect the content of studies available in the literature and as a result, findings may not reflect all studies conducted on these topics. Finally, the quality assessment in this study focused on study design and did not encompass many aspects of statistical analysis, an assessment of which may identify additional strengths and weaknesses of this literature.

## Conclusions

The findings and resources in this review provide methodological guidance to inform future policy relevant research on the impact of global trade and investment policies on tobacco, alcohol, diet, and related health outcomes. Future quantitative research on these topics should strive to clearly define exposures of interest and avoid conflating trade and investment; explore mechanisms of these relationships through analysis of mediating variables; and consider expanding the use of individual- and household-level data. Although not widely available for all exposures or outcomes, more sector-specific data should be creatively explored to pose more nuanced research questions and generate a better understanding of mechanisms for impact. Longitudinal analyses should test and adjust for autocorrelation and endogeneity and all analyses should present results from alternative statistical models and sensitivity analyses.

Measuring the impacts of global trade and investment on NCD-related health outcomes and risk factors in a rigorous and comparable way can support global policy action on NCDs. In particular, this will facilitate prospective assessment of potential health risks when designing new trade and investment agreements. This can also help identify strategies to preserve policy space to implement health-promoting policies that may have restrictive effects on trade or investment, and to uphold such policies if challenged in trade or investment disputes.

## Additional files

Additional file 1: Study details of 34 quantitative studies. (DOCX 47 kb)

Additional file 2: Data abstraction & quality assessment tool. (DOCX 40 kb)

## Abbreviations

BIT: Bilateral investment treaty
BMI: Body mass index
CSGR: Centre for the Study of Globalisation and Regionalisation
CVD: Cardiovascular disease
DESTA: Design of Trade Agreements project
FAO: Food and Agriculture Organization
FDI: Foreign direct investment
FTA: Free trade agreement
GDP: Gross domestic product
GNI: Gross national income
IMF: International Monetary Fund
OECD: Organization for Economic Cooperation and Development
PTA: Preferential trade agreement
RTA: Regional trade agreement
SAP: Structural adjustment program
SSB: Sugar-sweetened beverage
UN: United Nations
UNCTAD: United Nations Conference on Trade and Development
USDA: United States Department of Agriculture
WDI: World Development Indicators
WTO: World Trade Organization
