# Supplementary material for: Analyzing the impacts of global trade and investment on non-communicable diseases and risk factors: a critical review of methodological approaches used in quantitative analyses
Source: Global Health. 2018 May 24;14:53. doi: 10.1186/s12992-018-0371-8 (PMC5968469; doi:10.1186/s12992-018-0371-8)
Supplement: Supplementary file 1 — Study details of 34 quantitative studies. (DOCX 47 kb) [file 12992_2018_371_MOESM2_ESM.docx]

# **Analyzing the impacts of global trade and investment on non-communicable diseases and risk factors: a critical review of methodological approaches used in quantitative analyses**

Additional File 2: Study details of 34 quantitative studies

**Search terms**

TITLE field:

(review OR systematic OR synthesis)

AND

(trade OR investment OR liberalization OR liberalisation OR WTO OR RTA OR RTAs OR PTA OR PTAs OR globalization OR globalisation OR deregulation OR macroeconomic OR "structural adjustment" OR SAP)

AND

(health OR disease OR diseases OR NCD OR NCDs OR mortality OR "life expectancy" OR diet OR dietary OR nutrition OR nutritional OR tobacco OR smoking OR alcohol OR "risk factor" OR BMI OR obesity OR weight)

## Study details

| **Lead Author (Year)** | **Title** | **Countries** | **Years** | **Trade and investment indicators** | **NCD-related indicators** |
| --- | --- | --- | --- | --- | --- |
| Alam (2016) | Accounting for Contribution of Trade Openness and Foreign Direct Investment in Life Expectancy: The Long-Run and Short-Run Analysis in Pakistan | Pakistan | 1972-  2013 | (exports + imports) % of GDP, real inflows of FDI | Life expectancy at birth |
| Baker (2016) | Trade and investment liberalization, food systems change and highly processed food consumption: a natural experiment contrasting the soft-drink markets of Peru and Bolivia | Peru, Bolivia | 1999-  2013 | Dummies for pre-post: 1) ratification and 2) enforcement of Peru-US FTA | FDI inflows per capita; soft drink imports per capita; soft drink (various types) sales per capita; sugar from soft drinks per capita |
| Barlow (2017) | Impact of the North American Free Trade Agreement on high-fructose corn syrup supply in Canada: a natural experiment using synthetic control methods | Canada; 16 OECD countries (weighted average for synthetic control) | 1985-  2000 | Dummy for pre-post NAFTA | Supply of caloric sweeteners (kcal per capita) |
| Bergh (2010) | Good for Living? On the Relationship between Globalization and Life Expectancy | 92 countries (28 HICs, 41 MICs, 23 LICs) | 1970-  2005 | KOF Index (total, and disaggregated by economic/political/social); compare with CSGR Index | Life expectancy at birth (total and by sex) |
| Burns (2017) | Is foreign direct investment good for health in low and middle income countries? An instrumental variable approach | 85 LMICs | 1974-  2012 | FDI inflows as % of GDP (total and by primary/secondary/tertiary sector) | Life expectancy at birth; Adult mortality, per 10,000 adults |
| Bussmann (2009) | The Effect of Trade Openness on Women’s Welfare and Work Life | 134 countries | 1970-  2000 | total trade % of GDP | Female life expectancy at birth |
| Chaloupka (1996) | U.S. Trade Policy and Cigarette Smoking in Asia | 10 Asian countries (4 where Section 301 opened cigarette market & 6 with more protected cigarette markets) | 1970-  1991 | Dummy for pre-post Section 301 agreement (fraction to indicate portion of year in year agreement reached) | Per capita cigarette consumption; market share of U.S. cigarettes |
| Costa-Font (2014) | ‘Globesity’? The Effects of Globalization on Obesity and Caloric Intake | 26 countries (mostly high-income, few upper-middle income) | 1989-  2005 | KOF Index (economic, political, and social, disaggregated); compare with CSGR Index | % of population obese; Average caloric intake |
| DeVogli (2013) | The influence of market deregulation on fast food consumption and body mass index: a cross-national time-series analysis | 25 OECD countries | 1999-  2008 | Index of Economic Freedom | Adult (over age 20) mean BMI (total and by sex) |
| DeVogli (2014) | Economic globalization, inequality and body mass index: a cross-national analysis of 127 countries | 127 countries | 1980-  2008 | KOF Index (economic subdomain only); (imports + exports) % of GDP & FDI % of GDP - both subcomponents of economic index but also included separately in models | Mean BMI, adults of both sexes combined |
| Estime (2014) | Trade as a structural driver of dietary risk factors for noncommunicable diseases in the Pacific: an analysis of household income and expenditure survey data | 5 Pacific Island countries | single year between 2005-10, varies by country | imported foods as % of total in terms of 1) caloric intake and 2) expenditure | Expenditure on 'unhealthy' foods (% of total spending); 'unhealthy' foods caloric intake (% of total); National obesity rates |
| Goryakin (2015) | The impact of economic, political and social globalization on overweight and obesity in the 56 low and middle income countries | 56 LMICs | 1991-2009, selected years, by country (single year for 19 countries) | KOF Index (total, and disaggregated by economic/political/social) | Dummy for above normal weight (BMI>25) (women, aged 15-49) |
| Herzer (2015) | The long-run effect of trade on life expectancy in the United States: An empirical note | United States | 1960-  2011 | total trade % of GDP | Life expectancy at birth |
| Herzer (2012) | FDI and health in developed economies: A panel cointegration analysis | 14 high-income countries | 1970-  2009 | FDI % of GDP | Life expectancy at birth |
| Lee (2012) | South Korea’s entry to the global food economy: shifts in consumption of food between 1998 and 2009 | South Korea | 1998 & 2009 | "transition period when the Korean food system became open to global influences and trade" (not clearly defined indicator) | Consumption per capita and per consumer (for each of 53 food groups) |
| Levine (2006) | Does trade affect child health? | 129 countries | 1990 (or closest year available) | predicted (from gravity model) and actual total trade as % of GDP | Life expectancy at birth |
| Ljungvall (2013) | The freer the fatter? A panel study of the relationship between body-mass index and economic freedom | 31 high-income countries | 1983-  2008 (6 time points) | Economic Freedom of the World Index | Adult (over age 20) mean BMI (total and by sex) |
| Lopez (2016) | Is trade liberalization a vector for the spread of sugar-sweetened beverages? A cross-national longitudinal analysis off 44 low- and middle-income countries | 44 LMICs | 2001-  2014 | Applied tariff (MFN) (average for HS lines 2202 & 2009) | SSB imports per capita; SSB sales per capita |
| Martens (2010) | Is globalization healthy: a statistical indicator analysis of the impacts of globalization on health | 117 countries | 2007 | Maastricht Globalization Index (total and by each of five domains) | Probability of dying between age 15 and 60 per 1000 population |
| Miljkovic (2015) | Globalisation and Obesity | 79 countries | 1986-  2008 | trade % of GDP; FDI % of GDP; globalization social index (GSI); globalization economic index (GEI) (GEI dropped from final analyses) | % of adults obese, by sex |
| Mukherjee (2011) | Globalization and human well-being | 132 countries | 1970-  2007 (averages for every 5-yr period) | KOF Index (total, and disaggregated by economic/political/social) | Life expectancy at birth |
| Mwabu (1996) | Health effects of market-based reforms in developing countries | 103 countries (51 with SAPs, 52 without) | 1980-  1993 (predictor variables, 1980-91; outcome, 1993) | Dummy variable for successful implementation of structural adjustment reforms; additional dummies for the sector where reforms were implemented, e.g., agriculture, health | Difference between life expectancy at birth in 1993 and 80 (chosen as ideal life expectancy) |
| Nandi (2014) | Associations Between Macrolevel Economic Factors and Weight Distributions in Low- and Middle-Income Countries: A Multilevel Analysis of 200 000 Adults in 40 Countries | 40 LMICs | 2002 or 2003, varies by country | FDI % of GDP (converted to z-scores); Mean tariff rate, 1990-99 (converted to standardized scores) | Mean BMI among adults 18-65, by sex (country-level); Categorical BMI (overweight, normal, underweight) (individual-level) |
| Oberlander (2016) | Globalisation and national trends in nutrition and health - a grouped fixed effects approach to inter-country heterogeneity | 70 high- and middle-income countries | 1980-  2008 | KOF index (economic and social domains, separately) | Animal proteins, free fats, sugars (all in kcal per capita per day); diabetes prevalence; mean BMI |
| Owen (2007) | Is Trade Good for Your Health? | 219 countries | 1960-  1995 (5-year intervals) | Trade % of GDP; black market premium; Sachs-Warner Index (dummy variable); average imports weighted by trading partners' infant mortality rate | Life expectancy at birth (by sex) |
| Schram (2013) | Urbanization and International Trade and Investment Policies as Determinants of Noncommunicable Diseases in Sub-Saharan Africa | 48 Sub-Saharan African countries | 2008 | KOF Index (economic subdomain only) | Prevalence of overweight; prevalence of obesity; Proportion of deaths attributable to CVD |
| Schram (2015) | The role of trade and investment liberalization in the sugar-sweetened carbonated beverages market: a natural experiment contrasting Vietnam and the Philippines | Vietnam, Philippines | 1999-  2013 | dummy for pre-post WTO accession | SSB sales per capita (off-trade); growth rate of SSB sales (domestic companies); growth rate of SSB sales (foreign companies) |
| Stevens (2013) | Healthy trade: The relationship between open trade and health. | Not stated but about 99 countries, based on N in model output | 1970-2005 (5-yr intervals) | total trade % of GDP; dummy for U.S. FTA in force | Life expectancy at birth (by sex) |
| Stroup (2007) | Economic freedom, democracy, and the quality of life | 104 countries | 1980-2000 | Economic Freedom Index | Life expectancy at birth |
| Stuckler (2008) | Population Causes and Consequences of Leading Chronic Diseases: A Comparative Analysis of Prevailing Explanations | 56 countries | 1960-2000 | total FDI; total capital flows as % of GDP | CVD mortality rate (male); NCD mortality rate (male) |
| Stuckler (2012) | Manufacturing Epidemics: The Role of Global Producers in Increased Consumption of Unhealthy Commodities Including Processed Foods, Alcohol, and Tobacco | 50 LMICs | 1997-2010 | FDI % of GDP | Per capita sales of several categories of ultra-processed foods (in kg), tobacco (in USD value), alcohol (in USD value) |
| Tausch (2016) | Is globalization really good for public health? | 99 countries | 1970-2010 (5-yr intervals) | "actual flows of foreign capital" from KOF Index | Life expectancy at birth |
| Taylor (2000) | The impact of trade liberalization on tobacco consumption | 42 countries | 1970-1995 | (imports + exports) % of GDP | Cigarette consumption per capita |
| Umana-Pena (2014) | Assessment of the Association of Health with the Liberalisation of Trade in Services under the World Trade Organisation | 114 WTO members | 1995 & 2010 | Index of service sector liberalization; index of liberalization in the health and social services subsector | Life expectancy at birth; Change in life expectancy, 1995-2010; Ideal potential improvement in life expectancy, 1995-2010; Ratio of observed to ideal change in life expectancy |
