# Supplementary material for: Analyzing the impacts of global trade and investment on non-communicable diseases and risk factors: a critical review of methodological approaches used in quantitative analyses
Source: Global Health. 2018 May 24;14:53. doi: 10.1186/s12992-018-0371-8 (PMC5968469; doi:10.1186/s12992-018-0371-8)
Supplement: Supplementary file 2 — Data abstraction & quality assessment tool. (DOCX 40 kb) [file 12992_2018_371_MOESM1_ESM.docx]

# **Analyzing the impacts of global trade and investment on non-communicable diseases and risk factors: a critical review of methodological approaches used in quantitative analyses**

Additional file 1: Data abstraction & quality assessment tool

**Study Details: Data Abstraction**

1. What is the study design (e.g., natural experimental, cross-sectional analysis, longitudinal analysis)?
2. What countr(ies) are included?
3. What year(s) are covered?
4. What is the exposure of interest?
   1. What are the data source(s) for the exposure variable(s)?
   2. What indicator(s) are used for the exposure variable(s)?
5. What are the outcome(s) of interest?
   1. What are the data source(s) for the outcome variable(s)?
   2. At what level were data for the outcome variable(s) collected (e.g., individual, household, country)?
   3. What indicator(s) are used for the outcome variable(s)?
6. What type(s) of statistical test(s) and/or model(s) were used?

**Assessment of Study Quality**

1. Study Design
   1. Is the aspect of trade and/or investment (i.e., policy, liberalization, flows) that is being investigated clearly articulated in the research question(s)?
      1. Yes
      2. No
   2. Are trade and investment treated jointly or separately in the research question(s)?
      1. Only trade considered
      2. Only investment considered
      3. Both considered, jointly
      4. Both considered, separately
   3. Is the theoretical link between trade/investment and the outcome(s) described and supported with existing literature and/or a conceptual model?
      1. Yes, supported by literature only
      2. Yes, supported by conceptual model
      3. No
2. Indicators
   1. Do trade and/or investment indicator(s) align with the aspect of trade/investment (i.e., policy, liberalization, flows) examined in the study?
      1. Yes
      2. No
   2. If an index (e.g., globalization index) or broader macroeconomic policy (e.g., structural adjustment program) is used as the explanatory variable, is an attempt made to disaggregate trade/investment from other aspects of the index or policy?
      1. Yes
      2. No
      3. N/A
   3. If multiple trade/investment agreements are compared, is any adjustment made for variations in the depth or scope of agreements?
      1. Yes
      2. No
      3. N/A
   4. Do trade and/or investment indicator(s) reflect the whole economy or are they specific to one or more sectors most relevant to the outcome(s) examined?
      1. Economy-wide
      2. Sector-specific
3. Analysis
   1. What confounding, mediating, and moderating variables have been examined?
      1. Confounders:
      2. Mediators:
      3. Moderators:
   2. Did the authors report testing alternative statistical models and were any criteria provided for model selection?
      1. Yes
      2. No
   3. Is the potential for endogeneity or reverse causality mentioned and is there any description of measures taken to account for this?
      1. Yes, mentioned and addressed. Describe:
      2. Yes, mentioned, but no consideration in design or analysis
      3. Not mentioned
   4. If longitudinal data were used, was autocorrelation accounted for in statistical models?
      1. Yes
      2. No
      3. N/A
   5. Were any sensitivity analyses described to explore the robustness of findings to alternative methodological decisions and/or model specifications?
      1. Yes
      2. No
